# Curcumin-infused nanostructured lipid carriers: a promising strategy for enhancing skin regeneration and combating microbial infection

**DOI:** 10.1186/s12917-023-03774-2

**Published:** 2023-10-16

**Authors:** Ola Elkhateeb, Mohamed E. I. Badawy, Hossam G. Tohamy, Howaida Abou-Ahmed, Mahmoud El-Kammar, Hoda Elkhenany

**Affiliations:** 1 Department of Surgery, Faculty of Veterinary Medicine, Alexandria University, Alexandria, 22785 Egypt; 2 Department of Pesticide Chemistry and Technology, Faculty of Agriculture, Alexandria University, El-Shatby, Alexandria, 21545 Egypt; 3 Department of Pathology, Faculty of Veterinary Medicine, Alexandria University, Alexandria, 22785 Egypt

**Keywords:** Regeneration, Herbal extract, Curcumin, Nanoparticles, Wound healing, Antioxidant, Antimicrobial

## Abstract

**Background:**

Curcumin is a biomolecule that can be extracted from the *Curcuma longa* that has been shown to have the potential to aid skin wound healing. It has been studied for its anti-inflammatory and antioxidant properties, which may help to reduce swelling and promote tissue repair. However, curcumin has low solubility in water, which can limit its absorption and bioavailability. Encapsulating it in lipid nanoparticles may help to increase its absorption, leading to improved bioavailability.

**Methods:**

Curcumin-loaded nanostructure lipid nanocarriers (CURC-NLCs) were prepared and characterized. Also, the phenolic, flavonoid contents, antioxidant and antimicrobial efficacy against gram-positive and gram-negative bacteria were investigated. Furthermore, in vivo rabbit animal model was used to test its regenerative capacity and wound-healing efficiency.

**Results:**

The CURC-NLCs significantly increased the content of phenolic and flavonoid compounds compared to curcumin, resulting in a dramatic increase in antioxidant activity. CURC-NLCs also showed a potent inhibitory effect on Gram-positive, Gram-negative, and fungi, two times higher than curcumin. CURC-NLCs showed a higher potential to fasten the wound healing of full-thickness skin injuries as it resulted in 1.15- and 1.9-fold higher wound closure at the first week of injury compared to curcumin and control, respectively (p < 0.0001).

**Conclusion:**

These results suggest that CURC-NLCs have an excellent potential to promote skin regeneration, which could be attributed to its antioxidant and broad-spectrum antimicrobial effect.

## Background

Herbal remedies have been used for centuries to aid in the healing of wounds. Many herbs have been shown to have properties that may be beneficial for wound healing, including antimicrobial, anti-inflammatory, and antioxidant properties. Curcumin is a natural compound found in the spice turmeric, a member of the ginger family. It is the active ingredient in turmeric and is responsible for its yellow colour. The chemical structure of curcumin consists of a cyclic diarylheptanoid, which is a type of polyphenol. It has a chemical formula of C_21_H_20_O_6_ and is composed of three main functional groups: a benzene ring, a hydroxyl group, and a ketone group [1]. Curcumin has been shown to have the potential to aid skin wound healing. Although the precise mechanisms underlying curcumin’s wound healing properties remain a subject of ongoing research, several studies have suggested that it may operate through a range of mechanisms. In the initial stage of wound healing, known as haemostasis, curcumin’s antiplatelet and anticoagulant properties have been proposed to play a role. By inhibiting excessive platelet aggregation and promoting normal blood clotting, curcumin could contribute to efficient haemostasis, thus controlling bleeding at the wound site [2]. Following haemostasis, the inflammatory phase begins, characterized by the influx of immune cells to the wound area. Curcumin’s well-established anti-inflammatory capabilities make it a potential candidate for modulating the inflammatory response during wound healing. It has the capacity to suppress the release of pro-inflammatory cytokines and attenuate excessive inflammation [3–5]. During the proliferation phase, fibroblasts play a pivotal role in producing collagen, a fundamental component for tissue repair, while angiogenesis occurs. Curcumin’s ability to stimulate collagen production and promote angiogenesis has been reported, which may lead to accelerated tissue regeneration during this stage. In the final remodelling phase, the newly formed tissue undergoes further maturation and organization. Curcumin might influence this stage by promoting the alignment and remodelling of collagen fibers, which can enhance tissue strength and functionality [6, 7].

In parallel, it is crucial to recognize that delayed or defective wound healing is a complex medical issue influenced by various factors, including microbial infections. In many cases, microbial colonization of wounds can significantly impede the healing process, leading to chronic wounds or complications [8]. Consequently, addressing microbial infections within wounds assumes paramount importance in fostering effective wound healing. Remarkably, Curcumin has demonstrated its potential to inhibit the growth of certain types of bacteria [9].

However, the curcumin bioavailability, or the amount of the compound absorbed and becomes active in the body, is relatively low [10]. This is due to several factors, including its low solubility in water, its rapid metabolism and elimination from the body, and its poor absorption from the gastrointestinal tract [11]. To increase the bioavailability of curcumin, a number of strategies have been developed, including the utilization of nanoparticle technology. These approaches may help to increase the amount of curcumin that is absorbed and becomes active in the body [12]. Loading curcumin into lipid nanoparticles, or small particles composed of lipids, is a strategy developed to increase curcumin’s bioavailability. Several types of lipid nanoparticles have been used to deliver curcumin, including liposomes, solid lipid nanoparticles, and nanostructured lipid carriers. These particles vary in size, composition, and properties, and each type has advantages and disadvantages [13].

Nanostructured lipid carriers (NLCs) represent a versatile lipid nanocarrier system designed for the delivery of various compounds, including drugs, cosmetics, and nutraceuticals. NLCs are composed of a mixture of solid and liquid lipids, and they can be prepared in various shapes and sizes, depending on the specific application [14]. There are several advantages to using NLCs as a delivery system for compounds, including improved bioavailability, as NLCs may improve the bioavailability of the compounds they deliver by enhancing their solubility and increasing their absorption [15]. Notably, numerous studies have demonstrated the utility of NLCs in enhancing the bioavailability and therapeutic efficacy of curcumin. For instance, numerous studies have explored the use of NLCs to improve the oral bioavailability of curcumin, attributing the enhanced absorption to the NLC’s ability to protect curcumin from degradation in the gastrointestinal tract [16–18]. Additionally, NLCs loaded with curcumin have demonstrated enhanced skin permeation, making them promising candidates for topical applications [19–21]. Moreover, in-depth investigations into the formulation of CURC-NLCs have indicated that these carriers offer a sustained release of curcumin, extending its therapeutic effects [20, 22]. These findings collectively support the use of NLCs as a suitable vehicle for curcumin delivery, emphasizing their potential in enhancing curcumin’s therapeutic properties.

Beyond enhanced bioavailability, NLCs offer additional benefits. They can enhance stability by protecting the compound from degradation and increasing its stability [23–25]. NLCs also may release the compound in a controlled manner, which can help extend its therapeutic effect and reduce the frequency of dosing [26]. Notably, NLCs can be used to deliver a wide range of compounds, including drugs, cosmetics, and nutraceuticals [18, 27, 28]. Furthermore, NLCs can be easily prepared using simple and scalable manufacturing processes [29].

For all these reasons, we have selected NLCs to encapsulate curcumin for promoting skin regeneration. In addition, we hypothesized that CUR-NLCs would provide a broad-spectrum of the antimicrobial effect, which could further promote wound healing.

## Methods

### Materials

Curcumin (C1386), 2,2-Diphenyl-1-picrylhydrazyl (DPPH), and Folin-Ciocalteau phenol reagents were obtained from Sigma-Aldrich (St. Louis, MO, USA). Other chemicals include ascorbic acid, capric acid, glycerol mono-stearate, lecithin, sodium carbonate, tween 80, tannic acid, Carbopol-942, triethanolamine, propylene glycol, ethanol, and methanol were purchased from EL-Gomhouria Company (Alexandria, Egypt). In addition, xyla-Ject (xylazine hydrochloride) and ketamine hydrochloride (ET13L087-11) were obtained as anaesthetic drugs for surgery from ADWIA Co. (10th of Ramadan City) and Rotexmedica (Trittau, Germany), respectively.

### Preparation of CURC-NLCs

**Fig. 1:**
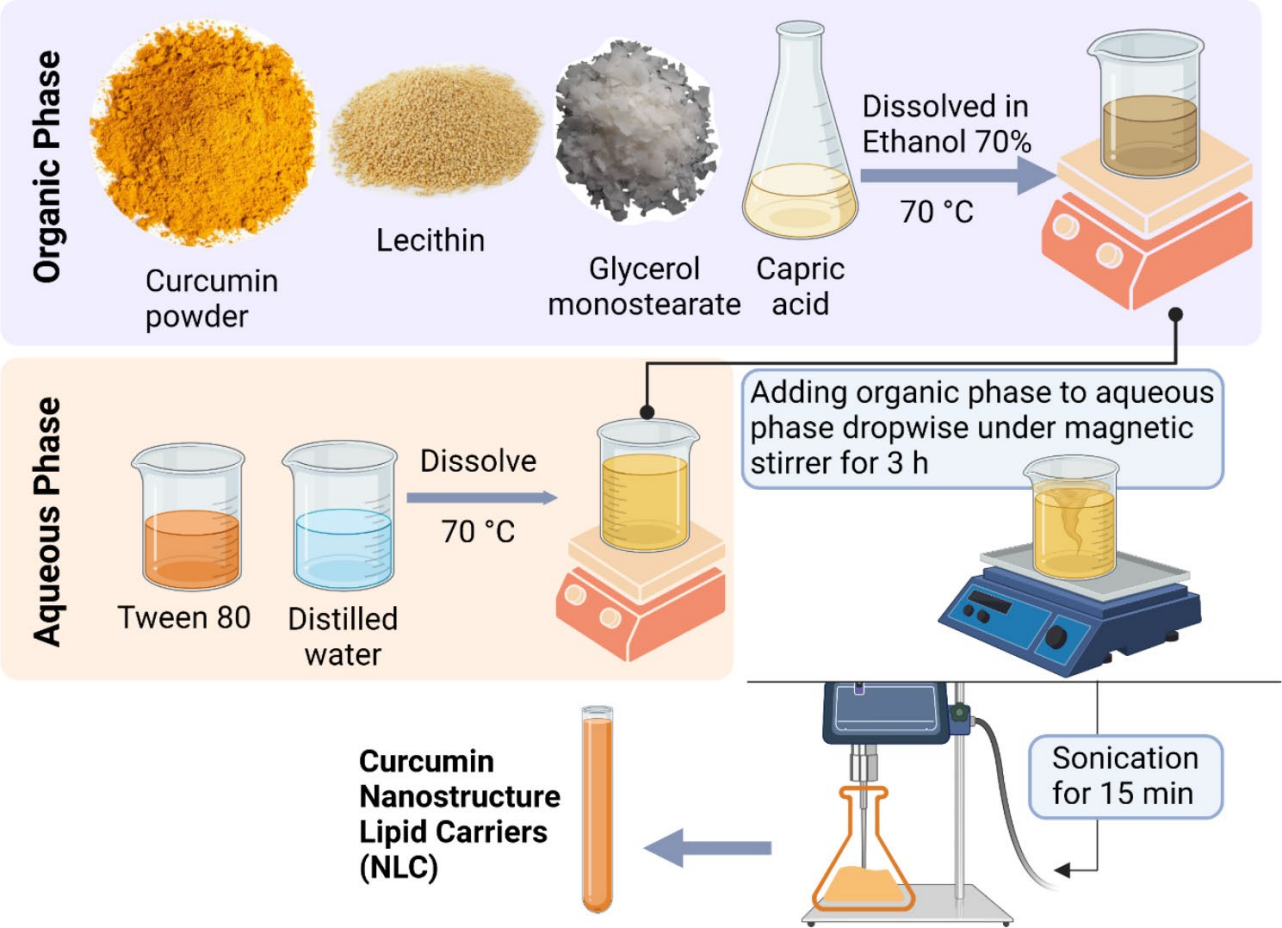
Preparation of Curcumin Nanostructured Lipid Carriers (NLCs) using emulsion-evaporation-solidification method. Created with BioRender.com

As previously described [26], the emulsion-evaporation-solidification method was used to prepare NLCs, as shown in Fig. 1, with some modifications. First, three concentrations (0.75%, 0.5%, and 0.25%, w/v) of curcumin were dissolved in 7% (w/v) glycerol mono-stearate (solid lipid), 3% (w/v) capric acid (liquid lipid), and 5% (w/v) lecithin in 15% (w/v) ethanol 70%. Next, this organic phase was heated to 70 °C, while the aqueous phase, consisting of 2% (w/v) tween 80 (surfactant) dissolved in 67.5% (w/v) distilled water, was also heated to 70 °C. Finally, under magnetic stirring, the organic phase was added to the aqueous phase dropwise at 70 °C for 3 h. The mixtures were then sonicated for 15 min at 7 kHz.

### Characterization of CURC-NLCs

The size of CURC-NLCs was analysed using a JEOL JSM-IT200 InTouchScope ^TM^ Scanning Electron Microscope (SEM Inc., Japan) and a JEOL JEM-1400 plus Transmission Electron Microscope (TEM Inc., Japan). Also, CURC-NLCs samples were analysed using photon correlation spectroscopy (Zetasizer Nano ZS, Malvern Instruments, USA). The measurements were carried out after dilution with distilled water and sonication. The polydispersity index (PDI) values were obtained using polystyrene cells at 25 °C and a refractive index (RI) of 1.330 (abs = 0.01) to measure the dispersion of the lipid nanoparticles. In addition, Zeta potential (ZP) was determined using zeta-dip cells at 12 runs to measure the charge on the surface of the nanoparticles and evaluate their physical stability [30]. Furthermore, entrapment efficiency (EE) and drug loading (DL) were determined using a modified version of the method described in Elkhateeb et al. [31]. CURC-NPs dispersion (0.1 mL) was centrifuged at 10,000 rpm for 20 min, and the supernatant was diluted with methanol (50:50). The resulting solution was then measured using a unico-1200 spectrophotometer at a wavelength of 323 nm. The EE (%) was calculated using the following equation:

$$\text{E}\text{E} \left(\text{\%}\right)= \frac{\text{A}\text{m}\text{o}\text{u}\text{n}\text{t} \text{o}\text{f} \text{d}\text{r}\text{u}\text{g} \text{a}\text{d}\text{d}\text{e}\text{d} - \text{A}\text{m}\text{o}\text{u}\text{n}\text{t} \text{o}\text{f} \text{d}\text{r}\text{u}\text{g} \text{i}\text{n} \text{t}\text{h}\text{e} \text{s}\text{u}\text{p}\text{e}\text{r}\text{n}\text{a}\text{t}\text{a}\text{n}\text{t}}{\text{A}\text{m}\text{o}\text{u}\text{n}\text{t} \text{o}\text{f} \text{d}\text{r}\text{u}\text{g} \text{a}\text{d}\text{d}\text{e}\text{d}}\times 100$$

The DL (%) was calculated using the following equation:

$$\text{D}\text{L} \left(\text{\%}\right)=\frac{\text{A}\text{m}\text{o}\text{u}\text{n}\text{t} \text{o}\text{f} \text{d}\text{r}\text{u}\text{g} \text{a}\text{d}\text{d}\text{e}\text{d} - \text{A}\text{m}\text{o}\text{u}\text{n}\text{t} \text{o}\text{f} \text{d}\text{r}\text{u}\text{g} \text{i}\text{n} \text{t}\text{h}\text{e} \text{s}\text{u}\text{p}\text{e}\text{r}\text{n}\text{a}\text{t}\text{a}\text{n}\text{t}}{\text{A}\text{m}\text{o}\text{u}\text{n}\text{t} \text{o}\text{f} \text{d}\text{r}\text{u}\text{g} \text{a}\text{d}\text{d}\text{e}\text{d}+\text{A}\text{m}\text{o}\text{u}\text{n}\text{t} \text{o}\text{f} \text{e}\text{x}\text{c}\text{i}\text{p}\text{i}\text{e}\text{n}\text{t}\text{s} \text{a}\text{d}\text{d}\text{e}\text{d}}\times 100$$

### Total phenolic content and antioxidant activity of curcumin and CURC-NLCs

The total phenolic in curcumin and CURC-NLCs at three different concentrations (25, 50 and 75 mg / 10 ml) were measured following the method described previously [31]. Furthermore, the antioxidant activity of curcumin and CURC-NLCs at the same concentrations mentioned above was measured using 2,2-Diphenyl picrylhydrazyl (DPPH) radical scavenging activity assay [32, 33].

### Encapsulation of curcumin and CURC-NLCs in Carbopol gel

To encapsulate the curcumin and CURC-NLCs, Carbopol-942, at a concentration of 5% was added to 7.5% of the curcumin or CURC-NLCs solution and stirred the mix. Triethanolamine (0.5 mL) and propylene glycol (a few drops) were added to neutralize the gel and improve its plasticity.

### Evaluation of the antimicrobial properties of the Carbopol encapsulated CURC-EXTR and CURC-NLCs

Carbopol-encapsulated curcumin and CURC-NLCs were tested for their microbial activity against the following bacterial strains (*Bacillus subtilis* ATCC 6633, *Escherichia coli* ATCC 25,922, *Salmonella spp*., *Staphylococcus aureus* ATCC 25,923, *Staphylococcus epidermidis*) and one fungal strain (*Candida albicans* EMCC 105). Strains were kindly provided by the American Type Culture Collection (ATCC, USA). The experiment was performed in triplicate, and the average values were recorded. The microbial culturing technique followed the previously described method in [31]. In addition, the ability of the curcumin or CURC-NLCs loaded gel to inhibit microbial growth was evaluated based on the inhibition zone diameter (IZD). As previously described, the minimal inhibitory concentration (MIC) was measured using the disc diffusion method for all diluted solutions [31, 34].

### Surgical animal model and treatment protocol

Eighteen male rabbits (New Zealand, weighing 1.9−2.0 kg) purchased from Faculty of Agriculture, Alexandria University. The animals used in this study were screened for any pre-existing health conditions and were acclimated for a period of 7 days prior to the start of the experiment. During this acclimation period, the animals were housed in the experimental animal facility under controlled environmental conditions of temperature and humidity, with a 12-hour light/dark cycle. The animals were provided with standard laboratory animal diet and water *ad libitum* and were observed daily for any signs of illness or distress. Rabbits were randomly divided into three groups carbopol alone, Curcumin @carbopol gel, and CURC-NLCs @carbopol gel, each group with six wounds. Full-thickness skin excisions with dimensions 3 × 3 cm were made on the dorsum of each rabbit. The skin excision surgeries were performed under general anaesthesia, with the animals sedated by intramuscular injection of xylazine (5 mg/kg) followed by ketamine (35 mg/kg). After surgery, the animals were injected with the analgesic drug meloxicam (0.2 mL). This surgical procedure was conducted according to the protocol approved by the Institutional Animal Care and Use Committee (AU-IACUC, AU01320190130108). The animals used in this study were not euthanized but were released at the completion of the study and that was deemed compatible with the ethical guidelines for animal experimentation. Prior to release, the animals were monitored to ensure they were in good health and able to thrive.

The different treatments were applied to the wounds seven days after surgery, as previously described [31]. After that, the wounds were followed up until complete healing (21 day). During the follow-up, multiple images were taken for wounds at days 0, 7, 14 and 21 in the presence of a refence object (well-known dimensions). These images were then analyzed using Image J software (ImageJ 1.53p, Java 1.8.0_112 (64-bit), USA) [35].

The % of wound healing was calculated using the following equation [36]:

$$\text{W}\text{o}\text{u}\text{n}\text{d} \text{h}\text{e}\text{a}\text{l}\text{i}\text{n}\text{g} \text{r}\text{a}\text{t}\text{e} \left(\text{\%}\right)=\frac{\text{I}\text{n}\text{i}\text{t}\text{i}\text{a}\text{l} \text{d}\text{a}\text{y} \text{w}\text{o}\text{u}\text{n}\text{d} \text{s}\text{i}\text{z}\text{e} - \text{S}\text{p}\text{e}\text{c}\text{i}\text{f}\text{i}\text{c} \text{d}\text{a}\text{y} \text{w}\text{o}\text{u}\text{n}\text{d} \text{s}\text{i}\text{z}\text{e}}{\text{I}\text{n}\text{i}\text{t}\text{i}\text{a}\text{l} \text{d}\text{a}\text{y} \text{w}\text{o}\text{u}\text{n}\text{d} \text{s}\text{i}\text{z}\text{e}}\times 100$$

### Histopathological examination

Tissue samples were collected from each animal (n = 5 from each group) using 10 mm biopsy punch (Integra® Miltex) under anaesthesia. After collecting tissue samples, the animals were carefully monitored during their recovery from anaesthesia to ensure their well-being. We provided appropriate post-operative care, including pain management and monitoring for any signs of distress or discomfort. Once the animals had fully recovered and were deemed to be in good health, they were safely released into their respective housing environments. Subsequently, the collected tissues were fixed in 10% neutral buffered formalin solution, embedded in paraffin wax, cut into five micron-thick sections and stained with haematoxylin-eosin (H&E) for examination by light microscopy [37]. To evaluate the histopathologic parameters of wound healing during 1st, and 3rd week was described as the following (0) absence of the lesion = 0%, (+) mild = 5–25%, (++) moderate = 26–50% and (+++) severe ≥ 50% of the examined tissue sections.

### Statistical analysis

In vitro and in vivo experimental data were collected from three independent experiments. The results were reported as the mean ± standard deviation (SD). Statistical analysis was performed using 2-way ANOVA followed by Šídák’s multiple comparisons test to compare different groups. GraphPad software version 8.0 (La Jolla, CA, USA) was used to analyze and generate a graphical representation of the data statistically. Differences were considered significant at * p < 0.05, ** p < 0.01, *** p < 0.001, and **** p < 0.0001.

## Results and discussion

Curcumin has been shown to have a number of potential skin health benefits, including antimicrobial, antioxidant, and wound-healing properties. Using NLCs could enhance the curcumin loading capacity and hence improve its delivery in topical applications in case of skin injuries. Herein, we have revealed the potential of NLCs to provide a protective layer around the curcumin, helping to preserve its activity and increase its stability, leading to increased skin benefits.

### Characterization of CURC-NLCs

**Fig. 2:**
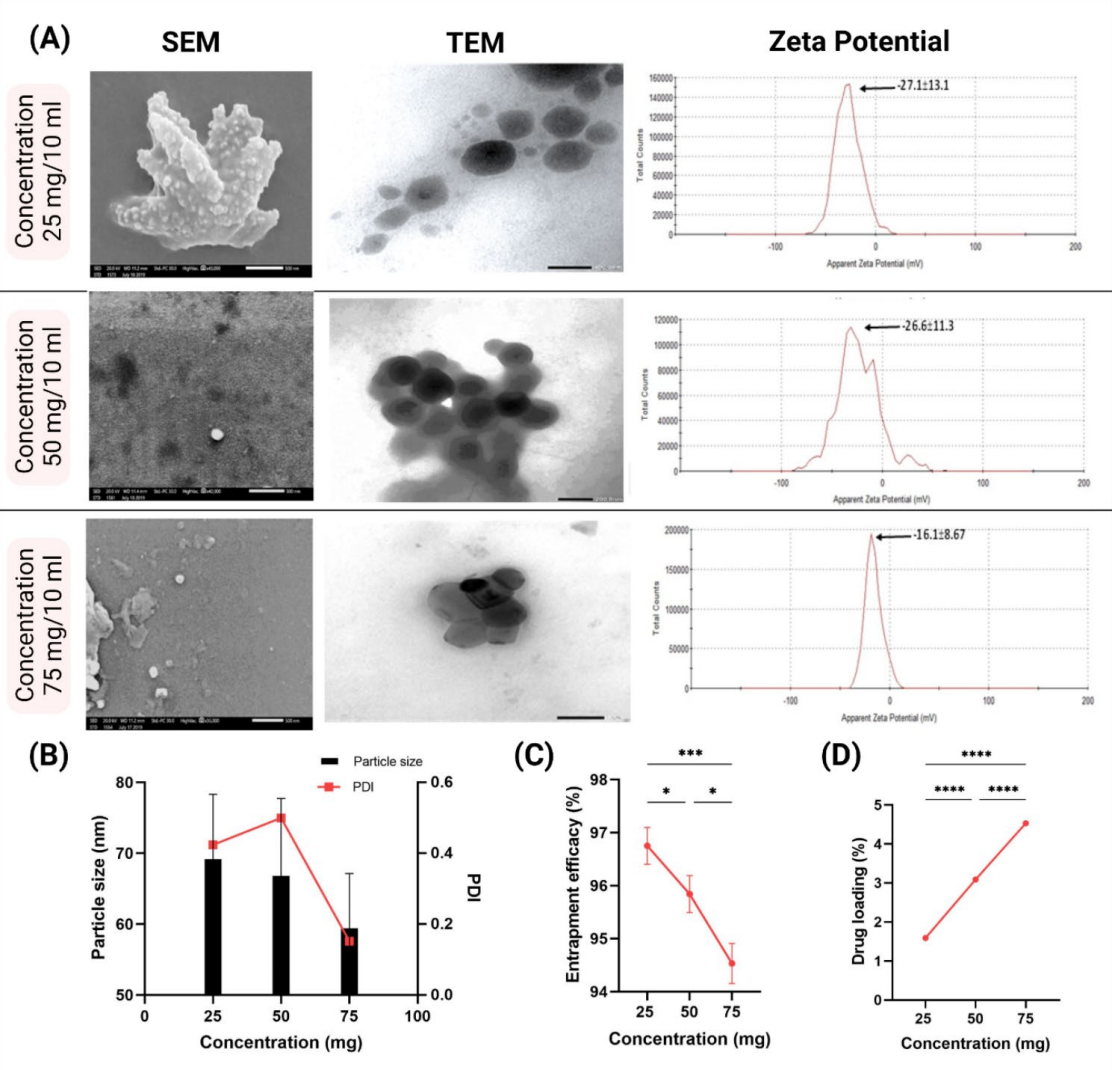
Characterization of CURC-NLCs at concentrations 25, 50 and 75 mg. (**A**) Scanning electron microscopy (SEM) Scale bar = 1 μm. Transmission electron microscopy (TEM), scale bar = 200 μm. Zeta Potential of CURC-NLCs. (**B**) Graph of particles size and Polydispersity index (PDI) of all tested concentrations. (**C** & **D**) Graphs show the Entrapment efficiency (EE), and Drug loading (DL) of CURC-NLCs formulations

SEM images showed irregular spherical particles for CURC-NLCs, as shown in Fig. 2A. The mean particle sizes of CURC-NLCs at concentrations 25, 50 and 75 mg / 10 ml was 69.17 ± 9.17, 66.81 ± 10.93 and 59.41 ± 7.72 nm (Fig. 2A). Furthermore, TEM images in Fig. 2B illustrated the morphology of the prepared nanoparticles, which were clearly compatible with the scanning microscopy results.

Photon correlation spectroscopy results for zeta potential and PDI for CURC-NLCs ranged between − 16.1 ± 8.67 to -27.1 ± 13.1 and 0.152 to 0.4 mV (Table 1; Fig. 2C). Of particular note, the formulation with a concentration of 75 mg/10 mL exhibited the highest zeta potential, affirming its long-term stability. Furthermore, our study demonstrated that the formulations achieved low PDI values, indicative of homogeneous particle size distribution (Table 1). This uniformity in distribution, especially notable in formulations with low PDI values.

**Table 1:** Characterizations of Curcumin nanostructured lipid carrier (NLC) formulations

Concentration (mg)	Particle size (nm) ± SD	ZP (mV) ± SD	PDI	EE (%) ± SD	DL (%) ± SD
25	69.17 ± 6.17	-27.1 ± 13.1	0.424	96.75 ± 0.35	1.59 ± 0.010
50	66.81 ± 10.93	-26.6 ± 11.3	0.5	95.84 ± 0.35	3.09 ± 0.014
75	59.41 ± 7.72	-16.1 ± 8.67	0.152	94.53 ± 0.38	4.53 ± 0.016

Scanning microscope measurements of particle size on scale 500 nm, entrapment efficiency (EE) and Drug loading (DL) of CURC-NLCs formulations

Our findings align with established literature on NLCs, which are known for their ability to encapsulate hydrophobic compounds like curcumin, resulting in the formation of nano-sized particles [38]. For instance, a study employing Stearic Acid and Caprylic/capric Triglycerides lipids in NLC preparation reported particle sizes ranging from 220 to 231 nm [39]. Another study using solid lipid glyceryl monooleate and Geleol, along with Olive oil as a liquid lipid, achieved particle sizes of 113.94 ± 11.3 nm and smaller PDI values (0.29 ± 0.05). In addition, studies employing lipids such as Precirol as a solid lipid, Capmul MCM as a liquid lipid, and Tween 80 and soya lecithin as surfactants reported a particle size of 146.8 nm with a zeta potential of -21.4 ± 1.87 [40].

### Entrapment efficiency and drug loading

This study analysed the EE and DL of CURC-NLCs using spectrophotometry at an optimal wavelength of 323 nm. The EE % results of CURC-NLCs at concentrations 25, 50 and 75 mg were 96.75 ± 0.35%, 95.84 ± 0.35%, and 94.5 ± 0.38%, respectively (Table 1). However, the DL % of the same concentrations were 1.59 ± 0.010%, 3.09 ± 0.014% and 4.53 ± 0.016%, respectively (Table 1). The results confirm that CURC-NLCs with a high drug concentration had a higher drug loading but a lower encapsulation efficiency than those with a lower drug concentration. Similarly, we have demonstrated previously that the highest concentration of propolis-NLCs possessed a high EE% and low DL% [31]. This data also matches the previous report by Chen et al. [41] which showed that increasing the drug-to-lipid ratio could improve DL capacity but decrease EE.

It has been demonstrated that EE and DL in NLCs are influenced by various factors, including drug solubility in lipids, miscibility of drug and lipid melts, solid lipid selection and its drug solubility, lipid polymorphic state, solid lipid ratio, lipid concentration, and the impact of surfactants. Notably, our formulation distinguishes itself by consistently achieving an impressive EE averaging at 95%, surpassing previously tested formulations. For instance, in the study conducted by Madane and Mahajan [40] study, an EE of 90.86% was reported, while Kamel et al. [22] research recorded an EE of 82.49%.

### CURC-NLCs exhibited higher total phenolics and antioxidant activity

Excess free radicals can lead to oxidative stress, causing damage to cellular components, including lipids, proteins, and DNA. This oxidative damage can impair the functionality of cells involved in wound healing, including fibroblasts and keratinocytes, thereby delaying the process [42, 43]. Moreover, oxidative stress can trigger chronic inflammation at the wound site, leading to the accumulation of pro-inflammatory cytokines and matrix metalloproteinases (MMPs). This sustained inflammatory response can hinder tissue regeneration and result in non-healing or chronic wounds [44, 45]. Therefore, strategies aimed at mitigating excessive free radical production or enhancing the antioxidant defense mechanisms can be beneficial in promoting efficient wound healing.

**Fig. 3:**
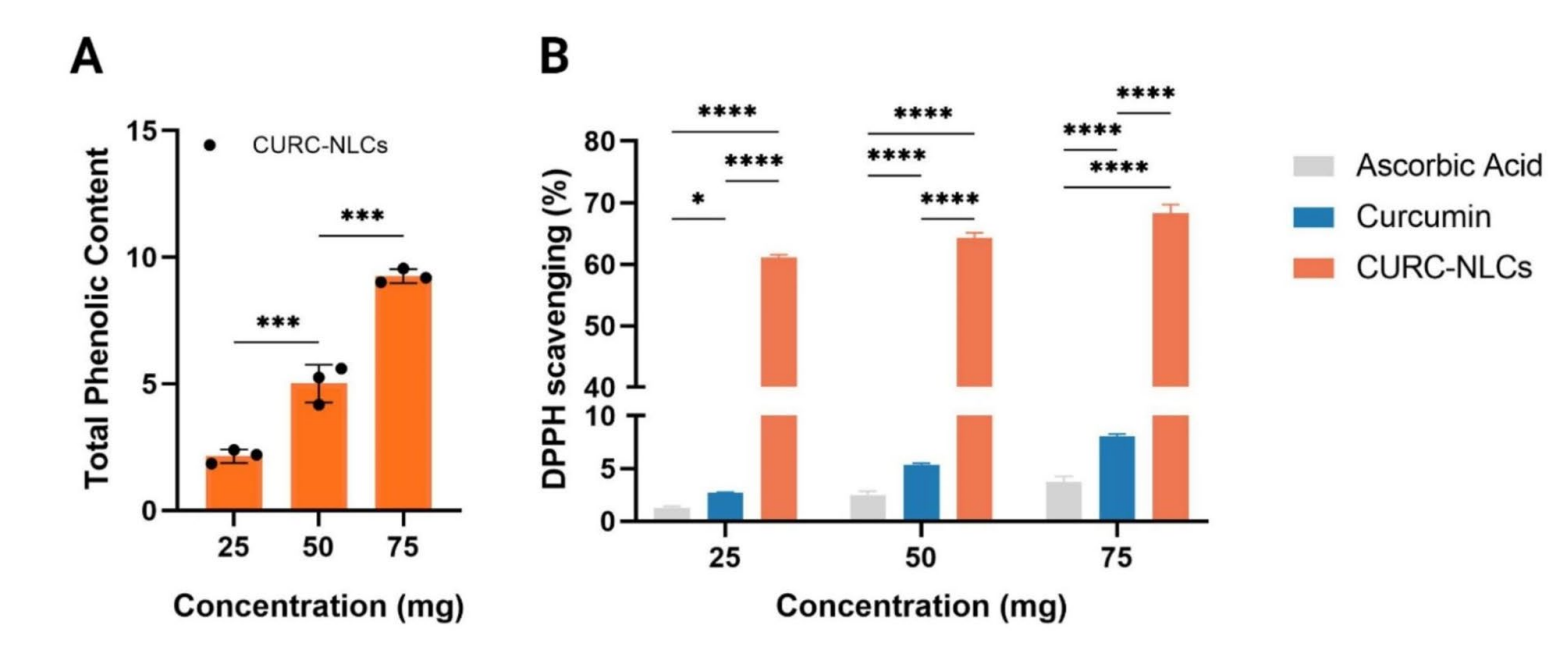
Phytochemical content of CURC-NLCs. (**A**) Total phenolic content of CURC-NLCs at concentrations 25, 50, and 75 mg. (**B**) The antioxidant activity of Curcumin and CURC-NLCs compared to ascorbic acid using DPPH scavenging activity test. Data represented as mean ± SD (*p < 0.05, ****p < 0.0001)

It is worth mentioning that herbal extracts are known for their robust antioxidant activity, a property closely linked to their phenolic and flavonoid contents [46]. These bioactive compounds have demonstrated a direct relationship with their ability to reduce oxidative stress and promote healing. Herein this study, the CURC-NLCs 75 mg had 9.26 ± 0.27 phenolic content compared to 6.33 ± 0.25 equivalent to tannic acid mg/g which was notably higher than the lower concentrations of 25 and 50 mg (*p* < 0.0001, Fig. 3A).

Curcumin has been shown to have antioxidant activity, which means that it can scavenge or neutralize harmful reactive oxygen species (ROS) that can damage cells and contribute to the development of various diseases. The antioxidant activity of curcumin may be due to its ability to donate electrons to ROS, rendering them inactive and preventing them from causing harm to cells [47]. Additionally, curcumin has been reported to stimulate the production of antioxidant enzymes within cells and activation of Nrf2-Keap1 signalling pathway, further contributing to its antioxidant activity [48].

Herein, we have investigated the DPPH scavenging capacity of CURC-NLCs to confirm the antioxidant activity of curcumin and CURC-NLCs. The results revealed that the CURC-NLCs possess a potent antioxidant activity compared to ascorbic acid (18-fold, *p* < 0.0001) and curcumin (~ 8.5-fold, *p* < 0.0001, Fig. 3B). A similar study has previously reported that CURC-NLCs can significantly enhance antioxidant ability, resulting in a remarkable 7-fold increase compared to CURC when assessed using the ABTS assay [21]. Another study conducted by Karimi et al. reported significantly higher antioxidant activity for CURC-NLCs compared to free curcumin extract using the DPPH assay [49]. Likewise, in our previous research involving the encapsulation of propolis in NLCs, we also documented significantly elevated antioxidant activity when compared to propolis extract [31, 50].

Hence, this study showed that CURC-NLCs have a stronger antioxidant effect than curcumin. This effect can be attributed to the high levels of phenolic and non-phenolic compounds entrapped in CURC-NLCs that can prevent oxidation reactions and attack free radicals. In addition, the transformation of curcumin into nanoparticles significantly improved its ability to scavenge free radicals compared to its native form.

The mechanism by which lipid nanostructures exhibit antioxidant activity may involve a combination of direct scavenging of ROS and indirect effects on the production of antioxidant enzymes within cells. For example, the presence of antioxidant lipids such as lecithin [51] and Tween 80 [52] within the nanostructures may directly scavenge ROS, while the delivery of curcumin well characterized antioxidants to cells would stimulate the production of antioxidant enzymes and enhance the overall antioxidant activity of the NLCs.

### CURC-NLCs performed significantly higher antimicrobial and antifungal activities

Microbes, particularly bacteria and fungi, can significantly influence the wound healing process. Infections resulting from these microorganisms can hinder and prolong the natural course of wound healing [53]. Specific bacteria, such as Staphylococcus aureus, Pseudomonas aeruginosa, and Escherichia coli, are known contributors to wound infections, producing toxins, biofilms, and inflammatory responses that disrupt the normal healing cascade [54, 55]. In this context, the antimicrobial properties of therapeutic agents become crucial in managing and expediting wound healing.

**Fig. 4:**
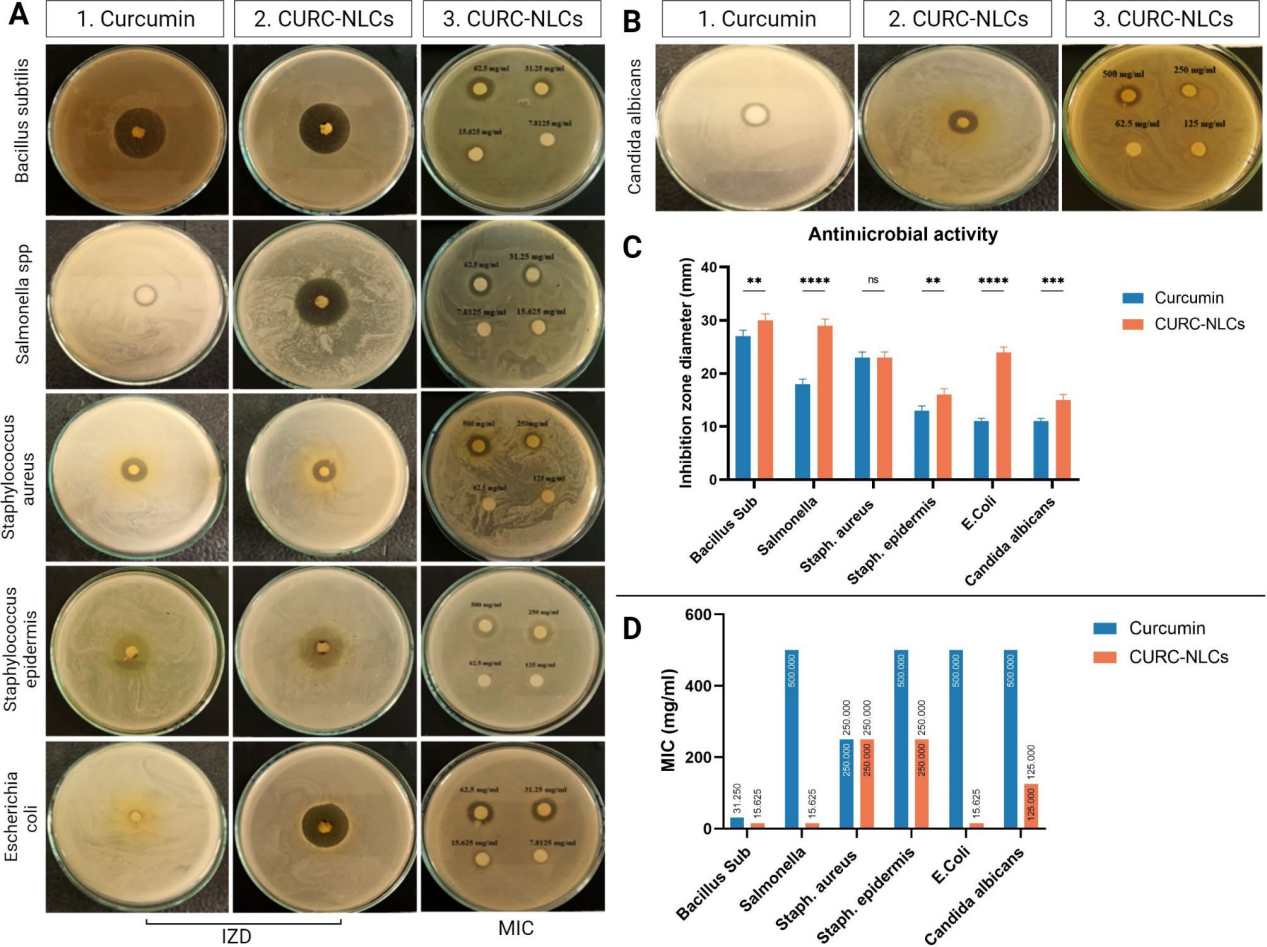
Assessment of the antibacterial and antifungal activity of Curcumin and CURC-NLCs. (**A**) Antibacterial activity of Curcumin and CURC-NLCs against different bacterial species as Bacillus subtilis ATCC 6633, Salmonella spp, Staphylococcus aureus ATCC 25,923, Staphylococcus epidermis, and Escherichia coli ATCC 25,922 using inhibition zone diameter (IZD) and minimal inhibitory concentration (MIC). (**B**) Antifungal activity of CURC-EXTR and CURC-NLCs against Candida albicans EMCC 105. (**C** & **D**) Quantitative analysis of IZD and MIC, data was represented as mean ± SD (^ns^*p* > 0.05, *p < 0.05, **p < 0.01, ***p < 0.001, ****p < 0.0001)

The antimicrobial activity of curcumin and CURC-NLCs against the tested microorganisms was determined by IZD and MIC, as shown in Fig. 4. The results of IZD confirms that the CURC-NLCs in carbopol gel displayed strong antimicrobial activity against *Bacillus subtilis*, *Escherichia coli*, *Salmonella spp*., *Staphylococcus epidermidis* bacterial strains and fungal strain (*Candida albicans*) compared to curcumin (Fig. 4; Table 2). Herein, both curcumin and CURC-NLCs exhibited similar effects against *Staphylococcus aureus* with nearly identical IZD measurements of approximately 23 mm. In contrast, a recent study by Hettiarachchi et al. reported recently that curcumin nanoparticles could enhance the antibacterial activity against *S. aureus* as confirmed by an increase in the IZD from 24.82 ± 0.54 mm in crude curcumin to 29.91 ± 0.53 mm in nano-formula [56]. On the other hand, our CURC-NLCs exhibited a notably more pronounced effect on E. coli, as evidenced by the substantial increase in IZD from 11 ± 0.78 to 24 ± 0.99. This aligns with a previous study where IZD for *E. coli* increased from 19.70 ± 1.18 to 24.58 ± 1.12 mm upon the use of curcumin nanoparticles [56].

**Table 2:** Antimicrobial activity evaluation of Curcumin and CURC-NLCs @Carbopol gels

Microbial Strains	Curcumin @Carbopol gel		CURC-NLCs @Carbopol gel	
	**MIC (** $\varvec{\mu }$ **g/mL)**	**IZD ± SD** **(mm)**	**MIC** **(** $\varvec{\mu }$ **g/mL)**	**IZD ± SD** **(mm)**
*Bacillus subtilis* ATCC 6633	31.25	27 ± 1.48	15.625	30 ± 1.56
*Escherichia coli* ATCC 25,922	500	11 ± 0.78	15.625	24 ± 0.99
*Salmonella spp*	500	18 ± 1.27	15.625	29 ± 1.27
*Staphylococcus aureus* ATCC 25,923	250	23 ± 1.41	250	23 ± 1.48
*Staphylococcus epidermis*	500	13 ± 1.13	250	16 ± 0.21
*Candida albicans* EMCC 105	500	11 ± 0.71	125	15 ± 0.14

It is also worth mentioning that the MIC of CURC-NLCs was significantly lower than that of curcumin, as evidenced by a reduction of almost half in the concentration needed to inhibit microbial growth in the epidermis of *Bacillus* and *Staph* (from 31.25 to 500 in curcumin to 15.6 and 250 in CURC-NLCs, respectively) (Fig. 4; Table 2).

Several studies have demonstrated that curcumin can inhibit the growth of a variety of bacteria, including *Escherichia coli*, *Salmonella typhi*, and *Staphylococcus aureus* [57]. In addition, it may also have antimicrobial activity against other types of microorganisms, such as fungi and viruses. The curcumin’s antimicrobial properties are believed to work in two ways: it blocks cell division through perturbing of the filamenting temperature-sensitive mutant Z (FtsZ) protein functions in the Z-loop and hence damages the bacterial membrane, and it inhibits SOS-response and hinder the quorum sensing system (QS) by reducing the biofilm production and weakening of QS dependant factors [58].

The high antimicrobial capacity of CURC-NLCs can be attributed to the loaded curcumin. Additionally, the NLCs components may have antimicrobial activity of their own, contributing to the overall effectiveness of curcumin in combating infections. For example, capric acid has been demonstrated to possess an antibacterial effect against *Propionibacterium acnes* [59]. Also, Lecithin was previously reported to enhance the antibacterial effect of Eugenol against *E. coli* [60]. Therefore, when curcumin is incorporated into NLCs, it may be more effectively delivered to the site of infection, leading to increased antimicrobial activity.

### CURC-NLCs enhanced skin regeneration

**Fig. 5:**
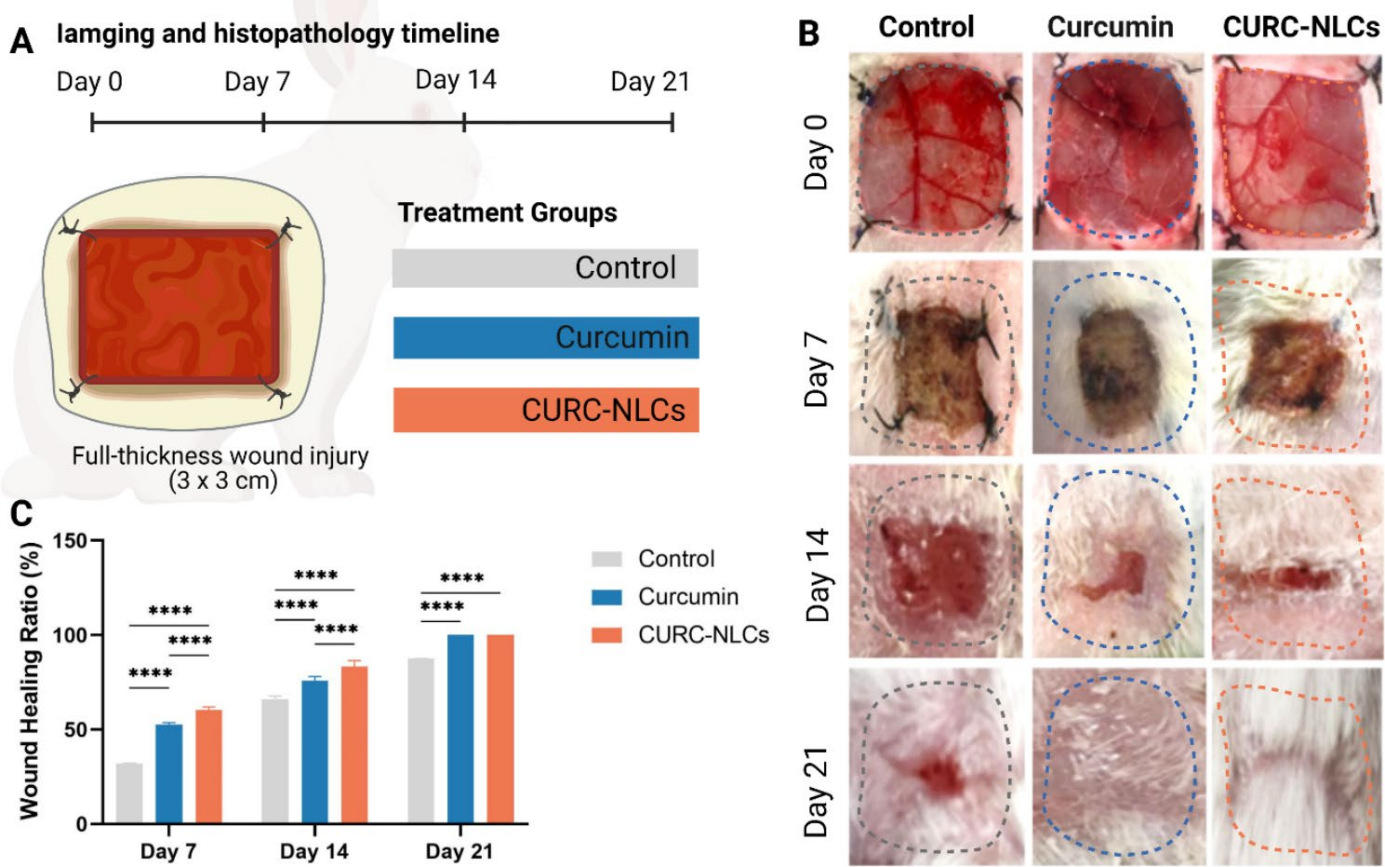
Wound healing evaluation after treated with Curcumin, CURC-NLCs in comparison to control group. (**A**) Schematic diagram showing the in vivo skin injury experiment design clarifying different treatment groups. Created with BioRender.com. (**B**) Representative images show wound dimensions after induction of injury (0 day) and then after 7, 14, and 21 days. (**C**) Qualitative analysis of the wound healing ratio in control, curcumin, and CURC-NLCs groups at days 7, 14 and 21 normalized to initial wound size at day 0, data represented as mean ± SD (****p < 0.0001)

CURC-NLCs-carbopol proved that treating wounds with nanoparticles promotes wound healing and reduces the epithelization period where the closure percentages were higher than the curcumin-carbopol treated wounds. They also revealed early stages of healing with 60.39 ± 0.04% wound closure on the 7th day, while curcumin-carbopol treated wounds were of 51.62 ± 0.09% closure. However, the carbopol treated wounds revealed a slow wound closure rate estimated by 31.85 ± 0.07% (Fig. 5). By day 14, CURC-NLCs-Carbopol treated wounds were significantly healing with 93.73 ± 0.04% wound closure, curcumin-carbopol and carbopol treated ones were closuring with 88.91 ± 0.03% and 65.22 ± 0.014%, respectively (Fig. 5). By day 21, there was complete closure of wounds at curcumin and CURC-NLCs groups however, control group was yet 87.49% wound closure.

The notable improvement in wound healing outcomes observed in our study can be attributed to the enhanced permeation and skin retention properties of CURC-NLCs, as corroborated by previously published ex-vivo skin permeation study. This study demonstrated a remarkable increase in permeation and skin retention when utilizing the CURC-NLC gel as compared to the free curcumin gel. This substantial enhancement in skin permeation and retention strongly suggests that CURC-NLCs excel at delivering curcumin precisely to the target site, thereby enabling a more prolonged and localized therapeutic effect [20]. Furthermore, our results align with in vitro findings that underscore the potential of CURC-NLCs to enhance wound healing. Specifically, CURC-NLCs have been shown to promote the proliferation of fibroblasts [21], key players in tissue regeneration, and enhance cell migration [61].

On the contrary, previous research has indicated an antimigratory effect associated with CURC-NLCs. Specifically, CURC-NLCs were found to reduce the migration and proliferation abilities of cells after 24 h, accompanied by noticeable morphological changes in keratinocytes when compared to control groups [21]. Additionally, investigations involving curcumin loaded onto poly (lactic-co-glycolic acid) nanoparticles have similarly revealed a potent inhibition of cellular activity in the HaCaT human keratinocyte cell line [62].

### Histopathologic findings and quantitative analysis

**Fig. 6:**
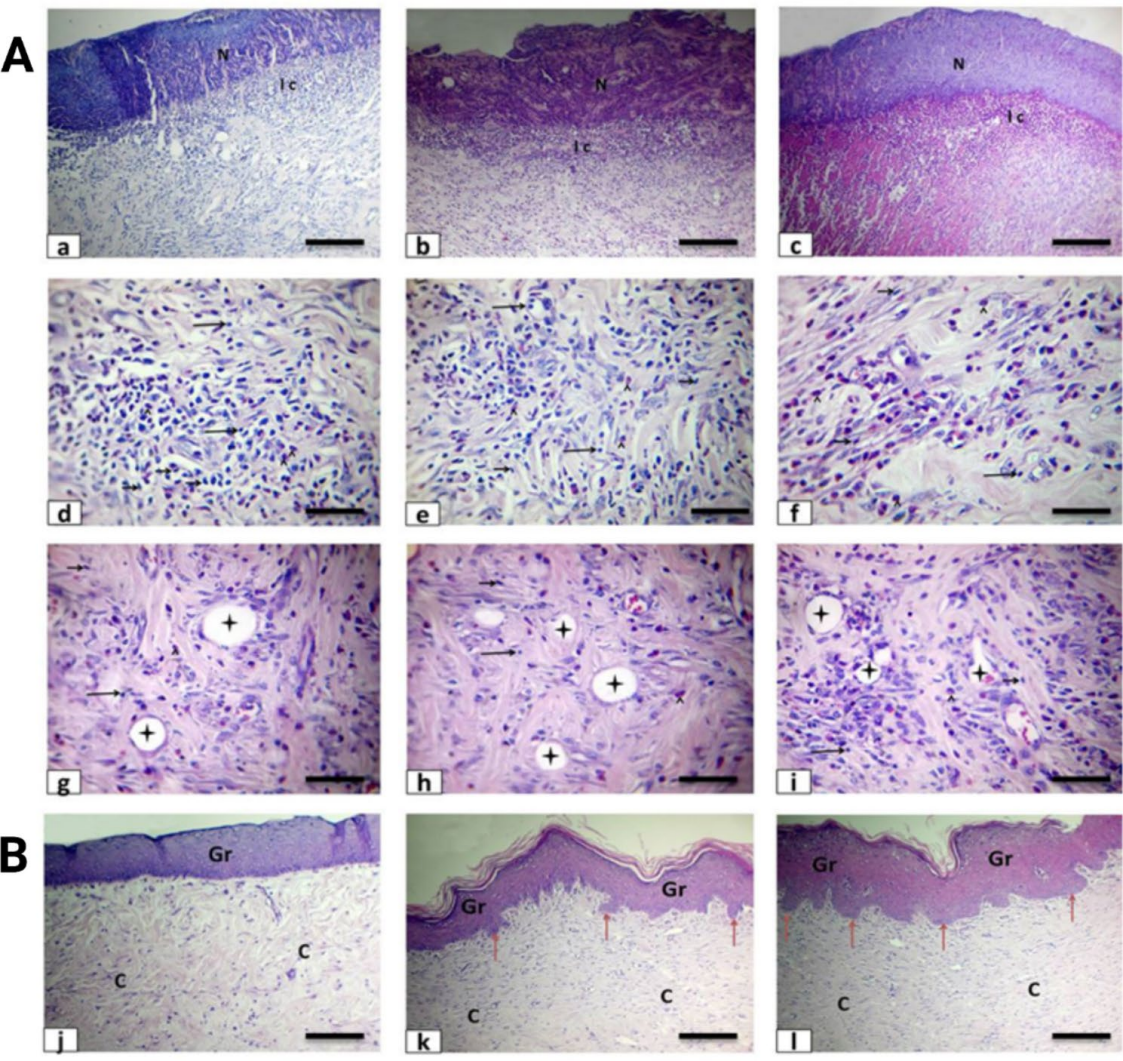
(**A**) photomicrograph of rabbit skin during 1st week stained by haematoxylin and eosin from the control (a, d & g), Curcumin (b, e & h), and CURC-NLCs (c, f & i) group showing necrotic layers (N), and interstitial inflammatory cell infiltration (IC) as mononuclear cell (long black arrows) and pleomorphic nuclear eosinophilic cell (arrowheads) with fibroblastic cell proliferation (short black arrows) beside neovascularization (asterisks). The bar = 100 μm for (a), (b), (c) and 50 μm for (d), (e), (f), (g), (h), (i). (**B**) Photomicrograph of rabbit skin during 3rd week stained by haematoxylin and eosin from the control (j), Curcumin (k), and CURC-NLCs (i) group showing epithelial regeneration and granular cell layer (Gr) with rete ridges (red arrows) mature collagen (C), hair follicle (F). The bar = 100 μm

Histopathological findings of injured rabbit skin of the control group during 1st week were scab formed by necrotic tissue remnants beside hyperaemic blood vessels in the dermis with severe haemorrhage and faint eosinophilic albuminous oedema. In addition, there was moderate acute interstitial inflammatory cell infiltration as pleomorphic nuclear eosinophilic cell and macrophage. However, in curcumin and CURC-NLCs group exhibited mild to moderate haemorrhage, proliferation fibroblastic cell and immature granulation tissues (Fig. 6A). In both the curcumin and CURC-NLCs treated groups, there was a reduction in the infiltration of macrophages and eosinophils compared to the untreated control group (Table 3). This observation is consistent with previous studies reporting the anti-inflammatory properties of curcumin and CURC-NLCs [63, 64].

**Table 3:** Histopathological scores for wound healing parameters in control, Curcumin and CURC-NLCs groups during the first and third week

Lesions/Groups	Incidence^1^ and Severity^2^ of Histopathological Lesions											
	**During 1st week**											
	**Control**				**Curcumin**				**CURC-NLCs**			
	-	+	++	+++	-	+	++	+++	-	+	++	+++
Scab formation	0	0	1	4	0	0	3	2	0	1	3	1
Edema	0	0	2	3	0	1	3	1	0	2	2	1
Hemorrhage	0	0	2	3	0	1	3	1	0	2	2	1
Macrophage	0	1	1	3	0	1	3	1	0	0	3	2
Eosinophil	0	1	2	2	0	2	2	1	0	1	3	2
Angiogenesis	0	1	2	2	0	1	1	3	0	0	2	3
Fibroblasts activity	3	2	0	0	3	1	1	0	2	2	1	0
	**During 3rd week**											
	**Control**				**Curcumin**				**CURC-NLCs**			
	-	+	++	+++	-	+	++	+++	-	+	++	+++
Fibroblasts activity	0	1	2	2	0	1	2	2	0	1	1	3
Re-epithelization	0	1	2	2	0	1	1	3	0	0	1	4
Rete ridges	3	2	0	0	0	1	1	3	0	0	2	3
Maturation of granulation tissue	0	1	2	2	0	1	2	2	0	1	1	3
Scar tissue formation	0	3	2	0	5	0	0	0	5	0	0	0

^1^Number of rabbits with lesions per total examined (5 rabbit per group)

^2^Severity of lesions was graded by estimating the percentage area affected in the entire section. Lesion scoring: (0) absence of the lesion = 0%, (+) mild = 5–25%, (++) moderate = 26–50% and (+++) severe ≥ 50% of the examined tissue sections

It is particularly noteworthy that the angiogenesis observed in the CURC-NLCs group was significantly more pronounced than in the other treatment groups, as indicated by a majority of samples receiving a high angiogenesis ranking (3 out of 5 ranked as +++). This heightened angiogenic response can be attributed to the potential enhancement of endothelial progenitor cells (EPCs). An intriguing study conducted by You et al. revealed that the administration of curcumin to diabetic mice resulted in a remarkable improvement in the migratory, angiogenic, and proliferative capacities of EPCs, coupled with a reduction in cellular senescence. Furthermore, this treatment was associated with a substantial upregulation in the expression of key angiogenic factors, including VEGF-A and Ang-1 [65].

The noticeable lesions during 3rd week in the control group were normal keratinized and epidermal layers covering the mature granulation tissue with mild formation of rete ridges. However, in curcumin and curcumin -NLCs group showed complete healthy epidermal regeneration in the form of thick keratin and granular epidermal layers covered with mature granulation tissue and collagen, with the wide formation of rete ridges, dermal glands structure and hair follicles (Fig. 6B). Incidence and severity of histopathological lesions of rabbit skin wound of all treatment groups were summarized in Table 3. Several in vivo studies have collectively shown that curcumin enhances cutaneous wound healing through various mechanisms. These include increased fibroblast proliferation, stimulated epithelial regeneration, facilitated tissue remodelling, promoted granulation and new tissue formation, as well as enhanced collagen deposition [66–68].

In a recap, the mechanism by which CURC-NLCs may improve full skin healing and be involved in all the skin structures may involve a combination of the following. First, the antioxidant availability of CURC-NLCs has been shown to have antioxidant activity, which may help to scavenge harmful ROS that can damage skin cells and contribute to the development of various skin conditions [69]. Second, angiogenesis, as evidenced by histological Sect. [65], can be a pivotal factor in skin regeneration. Angiogenesis is crucial for ensuring an adequate blood supply to the wounded area, facilitating nutrient and oxygen delivery, and promoting overall tissue repair. Additionally, CURC-NLCs exhibit antimicrobial activity that can address microbial infection which may hinder the wound healing process. By inhibiting the growth of pathogenic microorganisms, CURC-NLCs help create a more favourable environment for effective wound healing.

## Conclusion

CURC-NLCs were successfully prepared and characterized using SEM, TEM, ZP and various other analytical techniques, demonstrating good drug loading and drug entrapment efficiency. Our results suggest that CURC-NLCs exhibit strong potential to enhance the quality of skin healing and accelerate the healing process. This enhancement can be attributed to improved curcumin bioavailability, as well as the antioxidant and antimicrobial properties of CURC-NLCs.

## Data Availability

All data generated or analysed during this study are included in this published article.
